# Pretreatment of *Miscanthus* with biomass‐degrading bacteria for increasing delignification and enzymatic hydrolysability

**DOI:** 10.1111/1751-7915.13430

**Published:** 2019-05-29

**Authors:** Haipeng Guo, Yueji Zhao, Xuantong Chen, Qianjun Shao, Wensheng Qin

**Affiliations:** ^1^ School of Marine Sciences Ningbo University Ningbo 315211 China; ^2^ Department of Biology Lakehead University Thunder Bay ON P7B 5E1 Canada; ^3^ Faculty of Mechanical Engineering and Mechanics Ningbo University Ningbo 315211 China

## Abstract

Biomass recalcitrance is still a main challenge for the production of biofuels and high‐value products. Here, an alternative *Miscanthus* pretreatment method by using lignin‐degrading bacteria was developed. Six efficient *Miscanthus*‐degrading bacteria were first cultured to produce laccase by using 0.5% *Miscanthus* biomass as carbon source. After 1–5 days of incubation, the maximum laccase activities induced by *Miscanthus* in the six strains were ranged from 103 to 8091 U l^−1^. Then, the crude enzymes were directly diluted by equal volumes of citrate buffer and added *Miscanthus* biomass to a solid concentration at 4% (w/v). The results showed that all bacterial pretreatments significantly decreased the lignin content, especially in the presence of two laccase mediators (ABTS and HBT). The lignin removal directly correlated with increases in total sugar and glucose yields after enzymatic hydrolysis. When ABTS was used as a mediator, the best lignin‐degrading bacteria (*Pseudomonas* sp. AS1) can remove up to 50.1% lignin of *Miscanthus* by obtaining 2.2‐fold glucose yield, compared with that of untreated biomass. Therefore, this study provided an effective *Miscanthus* pretreatment method by using lignin‐degrading bacteria, which may be potentially used in improving enzymatic hydrolysability of biomass.

## Introduction

Various sources of lignocellulosic feedstocks have been regarded as emerging biofuels to partly replace the fossil fuels in the near future. However, their complicated cell wall structure makes the production of biofuels more difficult and costly due to the existence of biomass recalcitrance (Pauly and Keegstra, 2008). Among the three main polymeric components (cellulose, hemicellulose and lignin) constituting lignocellulosic biomass, lignin, which covers the cellulose and hemicellulose in various ways, is the most stable and complex polymer and acts as an obstacle in the enzymatic hydrolysis of cellulose (Mussatto *et al*., 2008; Lee *et al*., 2014). To make more cellulose and hemicellulose available before enzymatic hydrolysis, a pretreatment step is necessary to weaken and remove lignin (Lee *et al*., 2014). The lignin can be disrupted via physical, chemical and biological processes (Alvira *et al*., 2010). Various pretreatments methods through physical and chemical processes (Hu and Wen, 2008), alkaline and dilute acid (Si *et al*., 2015), organic solvent (Sun *et al*., 2000), steam explosion (Öhgren *et al*., 2007) and wet oxidation (Hendriks and Zeeman, 2009) are available to remove lignin from lignocellulosic biomass. However, high energy input, high costs of chemicals and increased environmental risk make these methods challenging to scale up (Millati *et al*., 2011). In addition, the lignin degraded by physical and chemical processes will release some inhibiting compounds, such as furfurals, 5‐hydroxymethyl furfurals and other volatile products, which inhibit the next stages of enzymatic hydrolysis and yeast fermentation (Hendriks and Zeeman, 2009; Camesasca *et al*., 2015). Organosolv pretreatment may cause severe damage like fire explosions in the absence of proper safety measures, although it can achieve a biomass fractionation to lignin, hemicellulosic sugars and a relatively pure cellulose fraction (Kumar and Sharma, 2017; Li *et al*., 2017). These are the disadvantages for large‐scale application.

Lignin removal via biological pretreatment has received increasing attention as an alternative to physical and chemical pretreatment for enhancing cellulose digestibility due to its environmental and economical benefits (Wan and Li, 2012; Sindhu *et al*., 2016). Among the various biological pretreatments adopted for lignin removal from lignocellulosic biomass, fungal pretreatments have been widely studied because fungi, especially white‐rot fungi, were thought to be excellent laccase and peroxidase producers (Wesenberg *et al*., 2003; Wan and Li, 2012). However, fungi usually grow slowly and require a long time to produce lignolytic enzymes like laccase, manganese peroxidase (MnP) and lignin peroxidase (LiP), which means that long pretreatment time is necessary for lignin removal by fungi (Leonowicz *et al*., 1999; Wan and Li, 2012). Moreover, large loss of cellulose and hemicellulose is another major weakness of fungal pretreatment, although remarkable enhancement of cellulose digestibility has been obtained from lignocellulosic biomass pretreated by white‐rot fungi (Wu *et al*., 2005; Lee *et al*., 2007).

A number of bacteria are able to produce various lignocellulolytic enzymes like endoglucanase (CMCase), xylanase and laccase (Bugg *et al*., 2011; Guo *et al*., 2017a). Of these bacteria, *Bacillus* sp., *Pseudomonas* sp., *Streptomyces* sp. and *Aeromonas* sp. strains have been reported to break down lignin (Bugg *et al*., 2011; Chang *et al*., 2014). *Bacillus* sp. CS‐1 isolated from forest soils was used to degrade up to 20% lignin in rice straw with remarkable laccase production (Chang *et al*., 2014). The laccase from *Bacillus licheniformis* showed strong oxidation capacity towards substrates 2,2‐azino‐bis (3‐ethylbenzothiazoline‐6‐sulphonic acid) (ABTS), syringaldazine, 2,6‐dimethoxyphenol and phenolic acids (Koschorreck *et al*., 2008). A soil bacterium, *Pseudomonas putida*, known as aromatic degrader, was capable of removing lignin from various lignocellulose biomasses accompanied by producing monocyclic phenolic products (Ahmad *et al*., 2010). However, the laccase production yields of bacteria are much lower than those of fungi and thus impeding their application in industry. In our previous study, it was found that the laccase production of bacteria can be significantly induced by lignocellulosic biomass, such as algae and *Miscanthus*. The high activity of laccase induced by *Miscanthus* may be due to the high components of total ester‐bound phenolics, which can stimulate the secretion of laccase (Guo *et al*., 2017b). In addition, the delignification efficiency of laccase can be largely improved by the laccase mediators, which can increase the oxidation of polymeric lignin by transferring hydrogen atoms (Baiocco *et al*., 2003; Yao and Ji, 2014).

Therefore, a two‐step pretreatment procedure was developed in this study. A low concentration *Miscanthus* biomass (0.5%, w/v) was first used to induce the production of laccase, and then, the crude enzymes of these bacteria induced by 0.5% *Miscanthus* biomass were directly mixed with equal volumes of citrate buffer and *Miscanthus* at solid concentration of 4% (w/v) to debilitate the lignin. The effects of two laccase mediators, 2,2‐azino‐bis(3‐ethylbenzothiazoline‐6‐sulphonic acid) (ABTS) and 1‐hydroxybenzotriazole (HBT), on the degradation of lignin were also determined in the process of pretreatment. Then, the pretreatment effect was evaluated by measuring the sugar released after enzymatic hydrolysis.

## Results

### Screening the Miscanthus‐degrading bacteria

**Figure 1:**
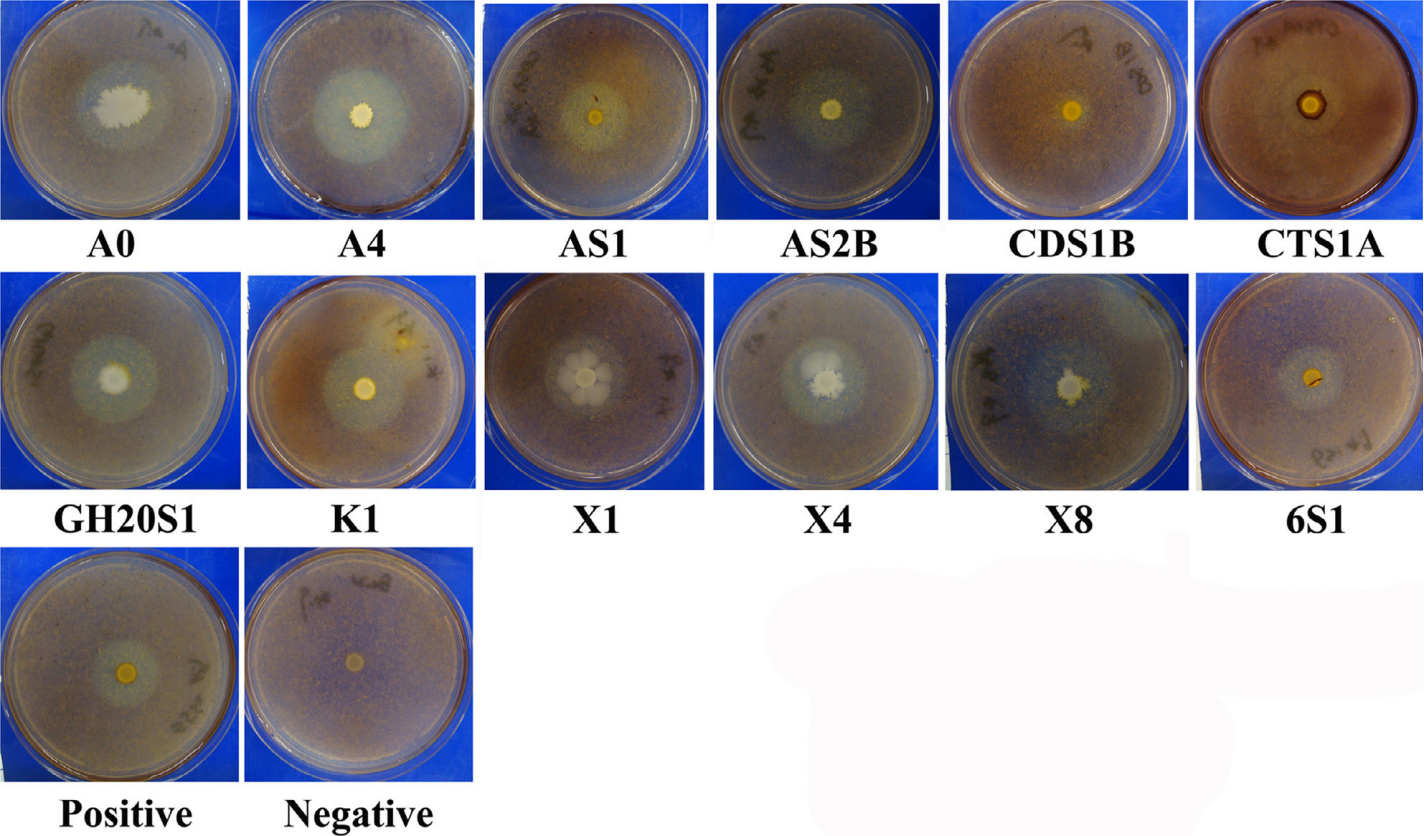
Visualization of the hydrolysis activities of *Miscanthus* biomass using twelve different bacteria strains and two control strains by staining with Gram's iodine solution. Positive: *C. xylanilytica*; Negative: *E. coli*.

Twelve bacterial strains isolated previously by Guo and colleagues (2017a,2017b), Maki and colleagues (2012) and Paudel and Qin (2015), including 7 *Bacillus*, 2 *Pseudomonas*, 1 *Exiguobacterium*, 1 *Aeromonas* and 1 *Raoultella* species (Table 1), were tested for the hydrolysis of biomass using *Miscanthus* as carbon source. All tested strains and positive strain *C. xylanilytica* showed a halo region in the plate after Gram's iodine staining, and nine strains (A0, A4, AS1, AS2B, GH2OS1, K1, X1, X4 and X8) showed bigger halo regions than that of *C. xylanilytica* (Fig 1). The hydrolytic potential of *Miscanthus* of each bacterial colony was further evaluated by calculating the square of the halo diameter: colony diameter ratio. The hydrolytic potential values of these strains ranged from 2.8 to 18.1, while only strains A4, AS1, AS2B, K1, X4 and X8 had higher hydrolytic potential than that of positive strain (hydrolytic potential value: 9.8) (Table 1). Therefore, these strains were selected for the next experiments.

**Table 1:** *Miscanthus* biomass hydrolytic abilities of twelve different bacteria and two controls

Isolates	Genus	Accession no.	Source	Halo diameter (D, cm)	Colony diameter (d, cm)	Hydrolytic potential (D/d)^2^
A0	*Bacillus*	KP974676	Paudel and Qin (2015)	3.25 ± 0.21b	1.45 ± 0.09a	5.02
A4	*Bacillus*	KX665584	Guo and colleagues (2017b)	3.43 ± 0.15ab	0.83 ± 0.02ef	17.1
AS1	*Pseudomonas*	HM063909	Maki and colleagues (2012)	2.85 ± 0.12c	0.67 ± 0.05 g	18.1
AS2B	*Exiguobacterium*	HM134063	Maki and colleagues (2012)	3.55 ± 0.06a	0.94 ± 0.06de	12.5
CDS1B	*Aeromonas*	FJ168772	Maki and colleagues (2012)	1.13 ± 0.1f	0.67 ± 0.03 g	2.8
CTS1A	*Bacillus*	KF482855	Maki and colleagues (2012)	2.34 ± 0.10e	1.10 ± 0.09c	4.5
GH2OS1	*Pseudomonas*	JQ320089	Maki and colleagues (2012)	3.38 ± 0.10b	1.32 ± 0.06b	6.5
K1	*Bacillus*	KP987117	Paudel and Qin (2015)	3.35 ± 0.13b	0.85 ± 0.04e	15.5
X1	*Raoultella*	KY290273	Guo and colleagues (2017a)	3.30 ± 0.18b	0.95 ± 0.07d	10.0
X4	*Bacillus subtilis*	KY327801	Guo and colleagues (2017a)	3.57 ± 0.10a	0.89 ± 0.04e	16.1
X8	*Bacillus*	KY941135	In this study	3.33 ± 0.10b	0.98 ± 0.05d	11.5
6S1	*Bacillus*	AB849115	Maki and colleagues (2012)	2.45 ± 0.26de	0.78 ± 0.04f	9.9
Positive	*C. xylanilytica*	AY303668	Rivas and colleagues (2004)	2.50 ± 0.08d	0.80 ± 0.02f	9.8
Negative	*E.coli* BL21	–	–	–	0.65 ± 0.03 g	–

Values represent mean ± SDs (*n* = 3). Different letters indicate a significant difference at *P *<* *0.05.

### *Activities of laccase secreted by* selected *bacteria*

**Figure 2:**
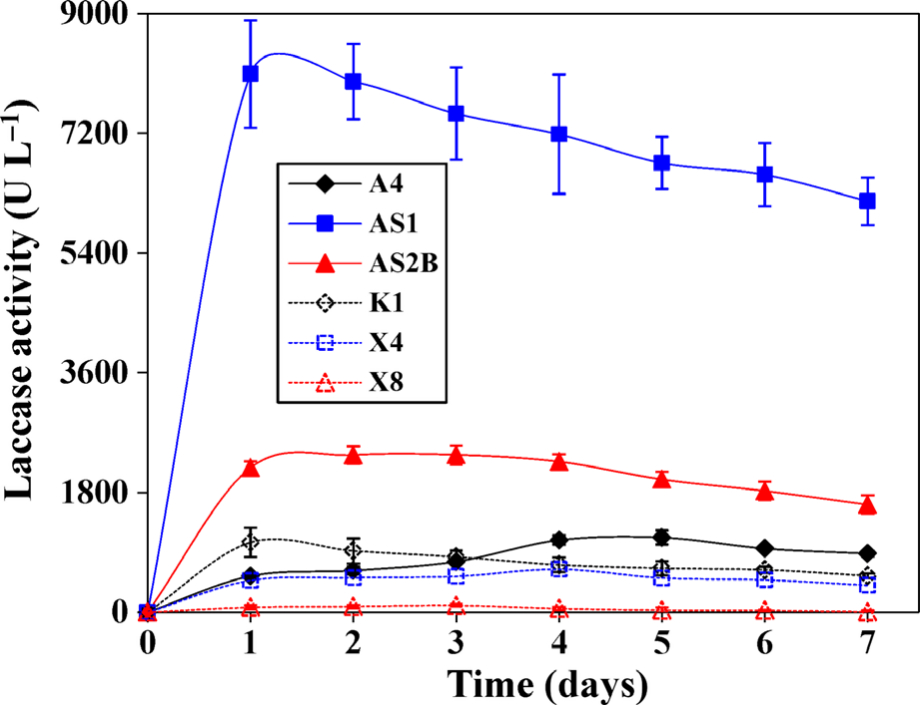
Laccase activities of different bacteria cultivated in the mineral salt medium containing 0.5% *Miscanthus* (w/v). Values represent mean ± SDs (*n* = 3).

The laccase production abilities were different among the six bacterial strains when *Miscanthus* was used as the sole carbon source (Fig. 2). Peak laccase activity was detected on the first day in strains AS1 (8091 U l^−1^) and K1 (1049 U l^−1^). Maximum activity occurred on the third day in strains AS2B (2365 U l^−1^) and X8 (103 U l^−1^), on the fourth day in stain X4 (646 U l^−1^), and on the fifth day in strain A4 (1122 U l^−1^) (Fig. 2).

### Effects of bacteria pretreatment on delignification

**Figure 3:**
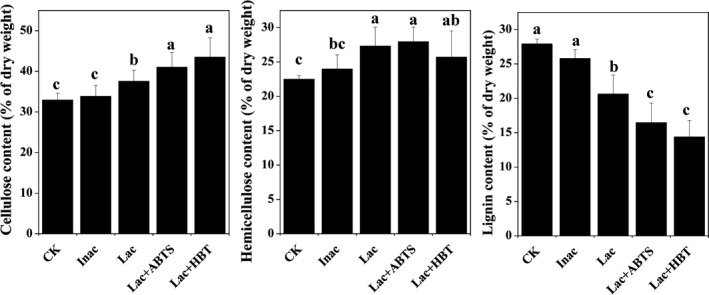
Variation of cell wall composition of *Miscanthus* after different bacteria pretreatment using ABTS or HBT as a mediator. (A) Cellulose content (*n* = 24); (B) hemicellulose content (*n* = 24); (C) lignin content (*n* = 24). ABTS: 2,2’‐Azino‐bis(3‐ethylbenzothiazoline‐6‐sulphonate), HBT: 1‐hydroxybenzotriazole. Different letters indicate a significant difference at *P *< 0.05.

The crude enzymes (Lac) produced by bacteria and two laccase mediators (Lac + ABTS and Lac + HBT) were tested in the enzymatic pretreatment of *Miscanthus* biomass. The crude enzymes autoclaved at 121°C for 25 min were used as a control (Inact). The native *M. sacchariflorus* used in this study mainly consisted of 32.9% cellulose, 22.5% hemicellulose and 27.9% lignin. After pretreatment of crude enzymes produced by the six bacteria, the cell wall composition of *Miscanthus* significantly differed with selective degradation of lignin, especially in the presence of two laccase meditators. Without laccase mediators, the lignin contents were 22.0, 19.5, 21.2, 16.1, 22.5 and 22.7% for Lac pretreatment of A4, AS1, AS2B, K1, X4 and X8 respectively. The most lignin was removed by Lac + HBT for all strains, and the lignin removal percentages were 47, 59.5, 42.7, 47.7, 52.7 and 41.2% for strains A4, AS1, AS2B, K1, X4 and X8 respectively. For Lac + ABTS pretreatment, strain AS1 degraded the most lignin of *Miscanthus*, with a lignin removal percentage of 50.9%, while the least lignin remove was found by X8 with the lignin removal percentage of 29.7%, compared with that of untreated *Miscanthus* (Table 2). Interestingly, the cellulose (Calculated as glucan) and hemicellulose (Calculated as xylan) contents were all significantly increased after pretreatment, compared with that of untreated and Inact‐treated *Miscanthus* (Table 2). The maximum cellulose content was obtained by Lac + HBT pretreatment of strain AS1, being 49.4% and 46.7% higher than those of untreated biomass and biomass treated by Inact respectively. The biomass pretreated by Lac + ABTS of X4 strain showed the highest hemicellulose content (30.1%) compared with that of untreated (22.5%) and Inact of X4 strain (23.5%). The almost no cellulose and hemicellulose loss were proved by the little sugar increase in the liquor after bacteria pretreatment (Table 2). Moreover, the addition of laccase mediators in all bacterial strains significantly changed the cellulose and lignin contents, while there were no significant effects to hemicellulose content compared with Lac pretreatment alone (Table 2 and Fig. 3). The Lac pretreatment alone included cellulose content ranging from 36.0% to 39.5%, hemicellulose from 25.6% to 30.1% and lignin from 16.1% to 22.7%, whereas using Lac + ABTS pretreatment, the ranges were 38.3–44.5%, 27.1–30.1% and 13.7–19.6%, respectively, and using Lac + HBT pretreatment, they were 39.3–49.3%, 21.1–28.9% and 11.3–16.4% respectively (Fig. 3A‐C).

**Table 2:** Composition changes of treated *Miscanthus* from different bacteria

Isolates	Methods	Glucan (%)		Xylan (%)		Lignin (%)
		Liquor	Solid	Liquor	Solid	
–	Untreated	–	32.9 ± 1.7	–	22.5 ± 1.5	27.9 ± 0.7
A4	Inactive	0.83	35.1 ± 4.4b	0.24	24.2 ± 2.7a	26.2 ± 1.1a
	Lac	1.08	38.1 ± 1.3ab	0.37	27.4 ± 3.9a	22.0 ± 2.6b
	Lac + ABTS	1.00	40.2 ± 2.6a	0.47	27.1 ± 1.2a	15.1 ± 2.1c
	Lac + HBT	1.04	41.1 ± 2.4a	0.54	26.7 ± 1.2a	14.8 ± 1.4c
AS1	Inactive	0.80	33.6 ± 2.7b	0.26	23.6 ± 1.0b	26.1 ± 0.6a
	Lac	1.09	36.2 ± 1.1b	0.47	25.6 ± 1.5b	19.5 ± 1.1b
	Lac + ABTS	1.07	44.5 ± 5.0a	0.48	27.0 ± 1.0a	13.7 ± 1.7c
	Lac + HBT	1.02	49.3 ± 3.4a	0.43	23.6 ± 1.3b	11.3 ± 3.4c
AS2B	Inactive	0.88	32.5 ± 2.8b	0.29	23.1 ± 2.6ab	25.8 ± 0.8a
	Lac	1.19	36.0 ± 3.6b	0.47	26.1 ± 1.9a	21.2 ± 0.9b
	Lac + ABTS	1.15	38.5 ± 3.0ab	0.42	27.3 ± 3.2a	18.9 ± 1.2bc
	Lac + HBT	1.03	41.4 ± 5.3a	0.49	21.1 ± 1.7b	16.0 ± 2.3c
K1	Inactive	0.85	33.5 ± 2.0b	0.27	23.5 ± 1.8b	26.4 ± 2.4a
	Lac	0.98	38.3 ± 1.0a	0.51	30.1 ± 2.8a	16.1 ± 1.5b
	Lac + ABTS	1.12	40.8 ± 3.6a	0.56	27.4 ± 1.9a	15.5 ± 2.5b
	Lac + HBT	1.17	41.5 ± 2.8a	0.61	26.3 ± 3.7ab	14.6 ± 3.5b
X4	Inactive	0.85	32.9 ± 2.8c	0.25	23.5 ± 1.6b	26.2 ± 2.4a
	Lac	1.13	36.6 ± 2.6c	0.38	25.7 ± 3.5a	22.5 ± 2.7b
	Lac + ABTS	1.04	43.3 ± 4.5b	0.49	30.1 ± 1.9a	15.7 ± 1.0c
	Lac + HBT	1.01	48.0 ± 4.5a	0.48	28.9 ± 2.0a	13.2 ± 1.1c
X8	Inactive	0.86	34.5 ± 2.4a	0.24	25.9 ± 2.6a	26.8 ± 1.8a
	Lac	1.02	39.5 ± 1.2a	0.43	28.7 ± 1.9a	22.7 ± 2.3b
	Lac + ABTS	1.09	38.8 ± 1.7a	0.45	28.3 ± 2.4a	19.6 ± 2.2bc
	Lac + HBT	1.10	39.3 ± 2.1a	0.51	26.0 ± 3.8a	16.4 ± 3.5c

Values represent mean ± SDs (*n* = 4). Different letters indicate a significant difference at *P *< 0.05.

### Effects of bacteria pretreatment on enzymatic hydrolysis

**Figure 4:**
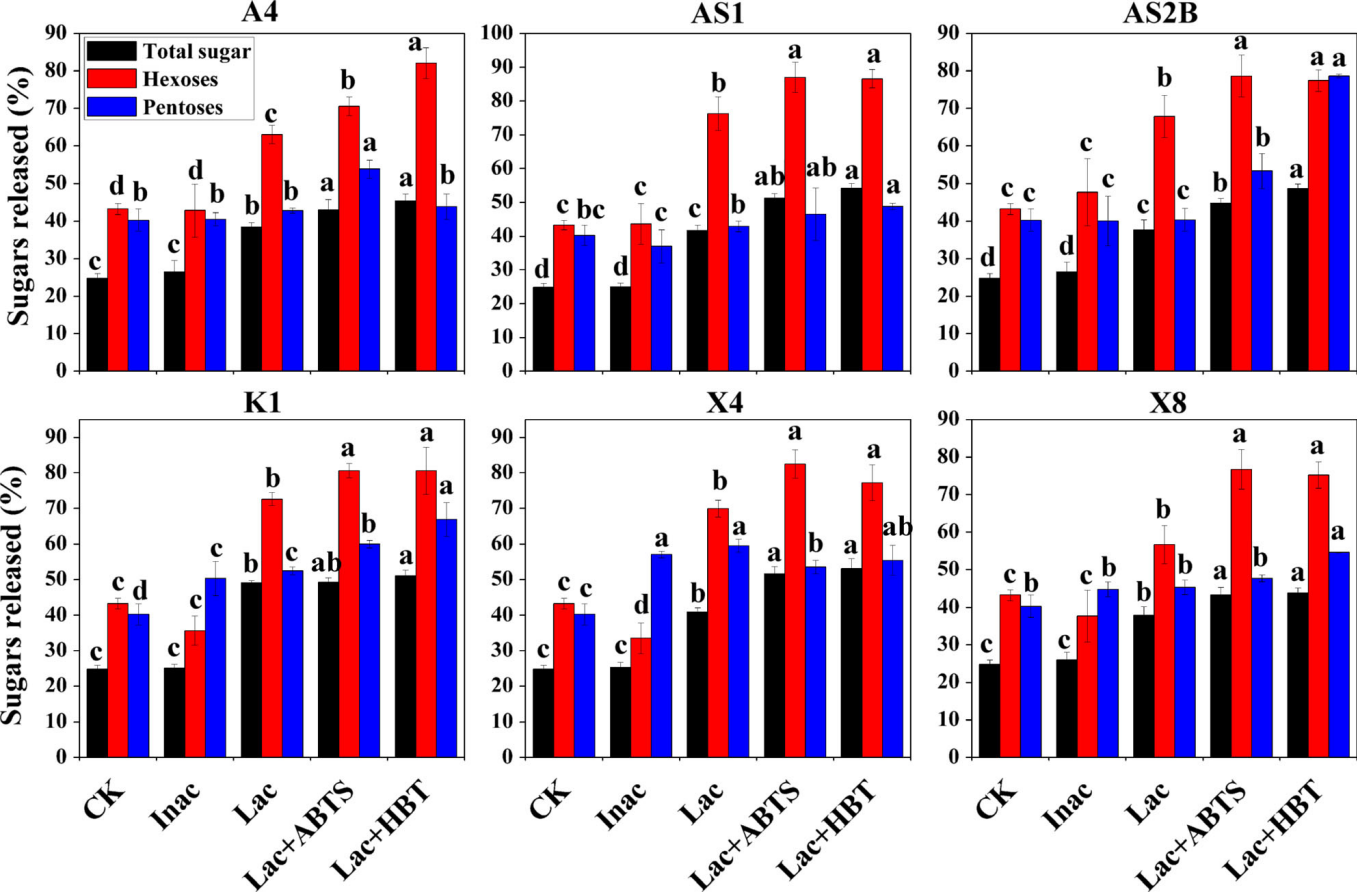
Biomass digestibility of *Miscanthus* after six different bacteria pretreatments. Total sugar yields (% of dry weight) released by mixed cellulase after pretreatment; Hexoses yields (% of cellulose) released by mixed cellulase after pretreatment; pentose yields (% of hemicellulose) released by mixed cellulase after pretreatment. Values represent mean ± SDs (*n* = 4). Different letters indicate a significant difference at *P *< 0.05.

To evaluate the effects of bacteria pretreatment on biomass digestibility, the sugar yield (hexoses and pentoses) released from pretreated *Miscanthus* after hydrolysing with a mixed cellulase cocktail (Celluclast 1.5L and Novozyme 188) was determined (Fig. 4). The hexoses released (% of cellulose), pentoses released (% of hemicellulose) and total sugar released (including hexoses and pentoses, % of dry weight) from untreated *Miscanthus* were 43.2, 40.3 and 25.4% respectively (Fig. 4). After enzymatic hydrolysis, the hexoses and total sugar released from pretreated *Miscanthus* by Lac, Lac + ABTS and Lac + HBT were all significantly increased (*P *< 0.05) in all strains, compared with that of untreated and corresponding pretreated *Miscanthus* by Inact. For each strain, the total sugar released from Lac + HBT was higher than that from Lac + ABTS and Lac. The highest total sugar yield was obtained by Lac + HBT of strain AS1 (54.2%), followed by Lac + HBT of strain X4 (53.1%) (Fig. 4).The hexose released from Lac + ABTS pretreated *Miscanthus* was higher than that of Lac + HBT and Lac in all strains except the strain A4, which obtained the peak hexoses yield by Lac + HBT (Fig. 4). The maximum hexoses were released by the Lac + ABTS of strain AS1 (87.0%; Fig. 4). For the release of pentoses, the maximum yields were harvested by Lac of strain X4 (59.5%), by Lac + ABTS of strain A4 (53.8%), by Lac + HBT of strains AS1 (48.8%), AS2B (78.7%), K1 (66.9%) and X8 (54.6%) respectively (Fig. 4).

### Correlation of lignin removal and sugars release and mass balance

**Figure 5:**
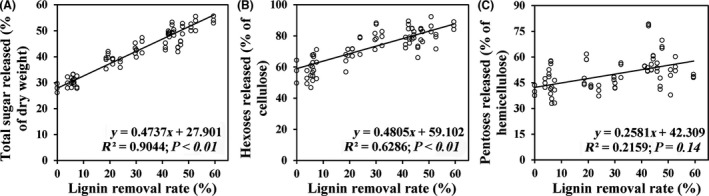
Correlation analysis between sugars released and lignin content after different bacteria pretreatment. (A) Total sugar released (*n* = 24); (B) hexoses released (*n* = 24); (C) pentoses released (*n* = 24).

We analysed the correlation between lignin removal and sugar released from six bacteria strains with or without mediators pretreated *Miscanthus* (Fig. 5). Significantly, a positive linear correlation was observed with correlation coefficient (*R* ^2^) values of 0.9044 (*P *< 0.01) and 0.6286 (*P *< 0.01) for the lignin removal and total sugar and hexoses yield released after bacteria pretreatment (Fig. 5A and B). However, there was no positive correlation between lignin removal and pentoses released by bacteria pretreated *Miscanthus* (*R* ^2^ = 0.2581; *P *= 0.14; Fig. 5C).

**Figure 6:**
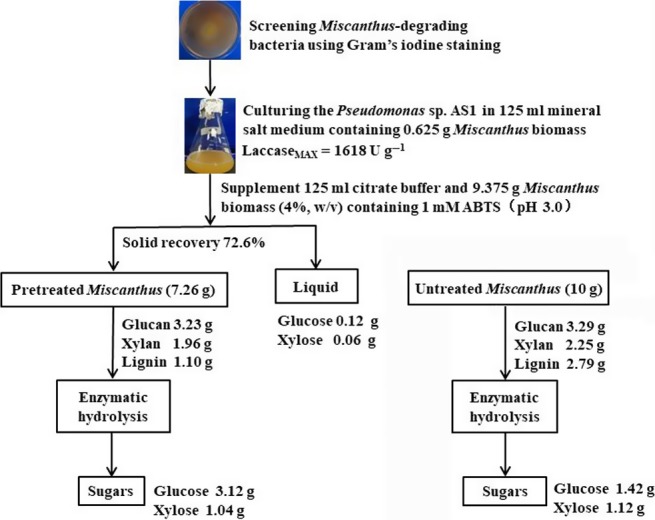
Flow chart of experiments investigated the effects of *Pseudomonas* sp. AS1 on laccase production, *Miscanthus* hydrolytic ability, lignin de‐polymerization and enzymatic hydrolysis in the presence of ABTS.

To further evaluate the effects of bacteria pretreatment and track carbohydrate degradation during the pretreatment process, the results obtained from laccase production, delignification and enzymatic hydrolysis were used to make a detailed mass balance using Lac + ABTS of strain AS1 (Fig. 6). The maximum laccase production in AS1 strain was 1618 U g^−1^ dry biomass. After Lac + ABTS pretreatment of strain AS1, the solid recovery was 72.6%, and the glucan content was unchanged (*P *> 0.05) compared with that of raw material, while the xylan and lignin content decreased from 2.25 g to 1.96 g and 2.79 g to 1.10 g per 10 g dry biomass compared with that of raw material respectively. After enzymatic hydrolysis, the glucose released from Lac + ABTS pretreated *Miscanthus* was 2.2‐fold higher than that of untreated *Miscanthus* (Fig. 6). The total monosaccharide yield was 4.34 g per 10 g dry biomass, which was a 1.7‐fold increase compared with that of untreated *Miscanthus* after enzymatic hydrolysis (Fig. 6).

## Discussion

### Miscanthus biomass is a good candidate for laccase production

Twelve biomass‐degrading bacteria (seven *Bacillus*, two *Pseudomonas*, one *Exiguobacterium*, one *Aeromonas* and one *Raoultella* species) were isolated on plates using carboxymethyl cellulose as the sole carbon source by our previously laboratory members (Bugg *et al*., 2011; Maki *et al*., 2012; Paudel and Qin, 2015). It has been reported that the degradation ability was extremely different using different carbon sources in both fungi (Olsson *et al*., 2003; Sipos *et al*., 2010) and bacteria (Waghmare *et al*., 2014; Mohammadkazemi *et al*., 2015). In this study, the degradation abilities of the twelve bacteria to *Miscanthus* biomass were determined using the Gram's iodine method, while only six bacteria strains (A4, AS1, AS2B, K1, X4 and X8) showed higher hydrolytic potential than that of the industrial strain *C. xylanilytica* when *Miscanthus* was used as the sole carbon source. To further evaluate the characteristics of these six strains, laccase activities were monitored in the mineral salt medium containing 0.5% (w/v) *Miscanthus*. The results showed the strains A4, AS1, AS2B, K1, X4 and X8 can efficiently secrete laccase into the medium, with the significantly different among the bacteria strains. Previous studies have shown the production of bacterial lignocellulolytic enzymes differed markedly using different carbon sources even if the same strain was used (Mohammadkazemi *et al*., 2015; Guo *et al*., 2017a). The maximum laccase activities induced by wheat bran in strains A4, AS2B, K1 and X4 were 246.7, 67.0, 82.4 and 137.0 U l^−1^, respectively (Guo *et al*., 2017a), and they were 501, 1149, 586 and 378 U l^−1^ in the presence of *Miscanthus* respectively. The lignocellulolytic enzyme activity produced by *Klebsiella* sp. PRW‐1 was higher when grass power and sugarcane were used as carbon source compared with that of other agricultural wastes like sorghum husks, corn straw and paddy straw (Waghmare *et al*., 2014). The biodegradation and enzyme production of lignocellulosic biomass by fungi and bacteria mainly depended on the porosity of biomass materials and crystallinity of cellulosic fibre (Zhang *et al*., 2006; Kumar *et al*., 2009). A series of lignocellulosic enzymes were produced by microorganisms using lignocellulosic biomass as carbon source, and certain lignocellulosic biomasses possess abundant soluble carbohydrates and enzyme synthesis‐related inducers, which can efficiently increase the production of lignocellulosic enzymes (Rosales *et al*., 2005; Singhania *et al*., 2010). Moreover, our results showed that in the presence of *Miscanthus,* the *Pseudomonas* strain AS1 specifically produced a large amount of laccase, which has been regarded as the main lignin‐degrading enzymes in the process of delignification of biomass (Martinez *et al*., 2009; Pollegioni *et al*., 2015).

### Delignification of *Miscanthus* and improved saccharification by bacterial pretreatment and laccase mediators

The polymers of lignin closely overlay the other polymers (mainly including cellulose and hemicellulose) in various ways in the lignocellulosics biomass. Lignin is very impervious and hard to degrade by enzymes or chemicals, and this is the main barrier to make cellulose and hemicellulose more susceptible to enzymatic hydrolysis in industrial processes (Brebu and Vasile, 2010). The degradation of lignin by using fungi has been well‐studied, especially by using white‐rot and brown‐rot fungi (Leonowicz *et al*., 1999; Guillén *et al*., 2005). But research about the degradation of lignin by using bacteria is scarce. In this study, six *Miscanthus*‐degrading bacteria were screened to degrade the lignin of *Miscanthus* biomass. The results showed that the crude enzymes produced by all the tested bacteria efficiently decreased the lignin content of *Miscanthus*, especially in the presence of two laccase mediators. The maximum lignin removal percentage (59.5%) was observed by the *Pseudomonas* sp. AS1 strain with the addition of 0.5% HBT (g g^−1^ dry biomass). Our previous study reported that the *Pseudomonas* sp. AS1 isolated from municipal waste showed good potential for black liquor decolourization and industrial degradation of lignocellulosic biomass (Maki *et al*., 2012). Moreover, the other four *Bacillus* sp. strains and one *Exiguobacterium* similarly reduced the content of lignin even if only their crude enzymes were used, which obtained the lignin removal percentage range from 18.6% to 42.3%. It has been reported that some of bacterial stains were able to degrade lignin via producing laccase and other lignin peroxidase (Bugg *et al*., 2011; Chang *et al*., 2014). The *Bacillus* sp. CS‐1 strain isolated from forest soils can degrade at least 61% alkali lignin within 48 h, and pretreatment using this strain followed by lactic acid bacteria removed 61.9% lignin of rice straw and enhanced cellulase performance (Chang *et al*., 2014). The *Pseudomonas* sp. LD002 and *Bacillus* sp. LD003 were isolated from beneath decomposing wood logs and exhibited good growth on lignin fractions and excellent dye‐decolourizing abilities (Bandounas *et al*., 2011).

The chemicals of ABTS and HBT have been regarded as the most efficient laccase mediators, which play as an electron carrier between laccase and oxidized substrate (Munk *et al*., 2015). In the current study, the average lignin contents of *Miscanthus* pretreated by six bacteria strains with two laccase mediators (ABTS and HBT) were 20.2% and 30.1% lower than that without laccase mediator. This was consistent with the higher lignin removal percentage obtained by laccase pretreatment in the presence of various laccase mediators (Rico *et al*., 2014; Rencoret *et al*., 2016). Furthermore, all bacteria pretreatment in the citrate buffer (pH 3.0) selectively degraded the lignin and improved the cellulose and hemicellulose contents of *Miscanthus*. The results were similar to that of pretreatment of feedstock with commercial laccase, which removed up to 50% of lignin and significantly increased the content of glucose and xylose in the presence of laccase mediators (Rico *et al*., 2014; Rencoret *et al*., 2016).

A previous study showed *Miscanthus* biomass saccharification largely depends on the effective lignin removal rather than the hemicellulose extraction under various alkali and acid pretreatments (Si *et al*., 2015). In this study, total sugar and hexoses released after enzymatic hydrolysis were significantly improved by all pretreatments and showed a positive correlation with the lignin removal percentage, which indicated that lignin ratio was the key factor that positively influenced *Miscanthus* digestibility after bacterial pretreatments. In addition, the maximum enzymatic digestibility (calculated as glucose released) from bacterial pretreatment was up to 87%, which was comparable with that of most fungal pretreatments, such as *Trameteshirsute* yj9 (73.99%; Sun *et al*., 2011) and *Pycnoporus* sp. SYBC‐13 (90%; Liu *et al*., 2013). Therefore, the bacterial pretreatment in this study might be more promising in the removal of lignin on *Miscanthus* compared with that of direct incubation biomass with fungi from the following three aspects: first, the laccase secreted by most of the tested bacteria in this study can rapidly reach the maximum after 1–3 days of incubation, which was easier to obtain crude laccases than most of fungi (Wesenberg *et al*., 2003; Liu *et al*., 2013). Second, even though the lower redox potential was found in most bacterial laccases (Bugg *et al*., 2011), the lignolytic enzyme activities induced by biomass in these bacteria were much higher than that in fungi. For example, the highest laccase activity of AS1 strain in this study was 1618 U g^−1^ dry biomass, while the maximum of it in reported fungi was 935.4 U g^−1^ dry biomass (Chang *et al*., 2012; Yang *et al*., 2012). Third, the process of lignin removal by fungal pretreatment was accompanied by large losses of cellulose and hemicellulose, which may be utilized as a carbon source to support fungal growth and metabolism (Wan and Li, 2010; Bugg *et al*., 2011).

## Conclusions

This study showed that the enzymatic digestibility of *Miscanthus* can be improved by laccase‐secreted bacteria. Six efficient *Miscanthus*‐degrading bacteria secreted abundant laccase when *Miscanthus* was used as sole carbon source. The crude enzymes induced by *Miscanthus* markedly decreased the content of lignin, especially in the presence of two laccase mediators, with the lignin removal percentage of 29.7–59.5%. After enzymatic hydrolysis, the glucose released from bacterial pretreated *Miscanthus* was 1.3–2.2‐fold higher than that from untreated biomass. These results will provide a two‐step pretreatment method to further studies on the use of laccase‐producing bacteria for bioenergy production.

## Experiment procedures

### Strains and culture conditions

The following twelve lignocellulose‐degrading bacteria, which were isolated and identified previously by our laboratory members, were used in this study (Table 1). The detailed information of *Bacillus* sp. A0 and K1 was described by Paudel and Qin (2015), *Bacillus* sp. A4, *Raoultella* sp. X1 and *Bacillus subtilis* X4 by Guo and colleagues (2017a) and *Pseudomonas* sp. AS1, *Exiguobacterium* sp. AS2B, *Aeromonas* sp. CDS1B, *Bacillus* sp. CTS1A, *Pseudomonas* sp. GH2OS1 and *Bacillus* sp. 6S1 by Maki and colleagues (2012). The strain *Bacillus* sp. X8 was isolated from forest soil (Thunder Bay, Ontario, Canada) and identified as *Bacillus* sp. X8 in this study. *Cellumonas xylanilytica* and *Escherichia coli* BL21 were used as positive and negative controls respectively. The strains and their corresponding accession number and source are shown in Table 1. All the strains were stored at −70°C. Prior to the experiments, all the strains were activated in Luria–Bertani (LB) medium at 37°C, with agitation at 200 rpm for 12 h. The bacteria were then cultured on a large scale in mineral salt medium (0.1% NaNO_3_, 0.1% K_2_HPO_4_, 0.1% KCl, 0.05% MgSO_4_, 0.05% yeast extract and 0.3% peptone) containing 0.5% (w/v) *Miscanthus sacchariflorus* (*M. sacchariflorus*) biomass for 7 days at 37°C, with agitation at 200 rpm. The production of laccase was monitored every day, and the culture medium from different strains with the highest laccase activity was directly used for *M. sacchariflorus* pretreatment.

### Screening and evaluation of Miscanthus‐degrading bacteria

To measure the hydrolysis activity of *M. sacchariflorus* by these bacteria strains, the strains were cultured on an agar plate using *M. sacchariflorus* as the sole carbon source according to our previous study (Guo *et al*., 2017a). Briefly, 5 μl of overnight‐grown each bacterial culture was dropped in an agar plate containing 0.1% NaNO_3_, 0.1% K_2_HPO_4_, 0.1% KCl, 0.05% MgSO_4_, 0.05% yeast extract, 0.3% peptone, 1.5% agar and 0.5% *M. sacchariflorus* biomass. All of the plates were incubated at 37°C for 48 h and flooded with Gram's iodine solution for 3–5 min. The diameters of the halo region (D) and bacterial colony (d) were then measured on a centimetre scale. The hydrolysis activity was calculated as (D/d)^2^, as described previously by Guo *et al*., 2017a.

### Determination of enzyme activities

For determination of laccase activities, 1 ml of cultures of each strain was collected after 1, 2, 3, 4, 5, 6 and 7 days of incubation and centrifuged at 12 000 *g* for 3 min. The supernatants were used as crude enzymes for enzyme activities analysis. The activities of laccase were measured according to the description of Guo and colleagues (2017a). Briefly, 200 μl of reaction solution including 20 μl diluted crude enzyme and 20 μl of 20 mM ABTS in a 0.1 M citrate buffer (pH 3.0) was incubated at 40°C for 3 min. Then, the optical density (OD) was measured at 420 nm (*ε*_420_ = 36 000 M^−1^ cm^−1^) using a Microplate Spectrophotometer (Epoch; Bio Tek Instruments, Winooski, VT, USA). One unit of laccase activity was defined as the amount of enzyme required to oxidize 1 μmol of substrate per min.

### Inoculum preparation and pretreatment

The screened six *Miscanthus*‐degrading bacteria (A4, AS1, AS2B, K1, X4 and X8) were firstly cultured in 125 ml mineral salt medium containing 0.625 g (0.5%, w/v) *M. sacchariflorus* biomass to produce laccase at 37°C, with agitation at 200 rpm. At the day with the highest laccase activity in the culture medium, equal volumes of citrate buffer (pH 3.0) and another 9.375 g *M. sacchariflorus* biomass were added to the culture medium to make the final biomass ratio at 4.0% (w/v). The final pH was adjusted to 3.0 using 1M hydrochloric acid. Then, the mixture was saturated with oxygen by bubbling air for 20 min. Tetracycline (40 mg l^−1^) was added into the mixture to control the growth of bacteria. For the effects of laccase mediators (HBT and ABTS) on the delignification of *Miscanthus* biomass, 0.1 mM ABTS and 5% HBT (g g^−1^ dry weight biomass) were added to the culture. Bacterial culture autoclaved at 121°C for 25 min was used as a control. The pretreatment was performed at 37°C, with agitation at 200 rpm. After 96 h of pretreatment, the samples were washed with distilled water five times to remove the bacterial cells through a double‐layered muslin cloth with the meshes of 300. Then, solid residues were oven dried at 50°C until constant weight and used for cell wall composition analysis and subsequent enzymatic hydrolysis.

### Plant cell wall composition analysis

The cellulose and hemicellulose contents were measured according to the method of Guo and colleagues (2017c). Briefly, the sample (0.1 g) was extracted twice with 1.5 ml hot water, once with 1.5 ml absolute ethanol and once with 1.5 ml acetone at 65°C for 30 min. Then, the sediment was air‐dried in a chemical hood for 2 days, and the dry samples were hydrolysed with 1 ml of 72% (w/w) sulphuric acid (H_2_SO_4_) at 30°C for 1 h. The hydrolysates were diluted to 3% sulphuric acid by adding deionized water and autoclaved at 121°C for 1 h. After cooling down, the mixture was filtered through a pre‐weighted glass microfilter. The solutions were used to determine the contents of hexose and pentose using the anthrone‐sulphuric acid method and orcinol‐hydrochloric acid method respectively. The filter and solids were dried at 105°C until constant weight and weighed, and then, the solids were ashed at 575°C for 3 h and weighted again. The Klason lignin contents were calculated as the weight of the dry solids minus that of the ash as a percentage of the weight of the initial dry weight of sample.

### Determination of total hexoses and pentoses

The total hexose and pentose contents were determined according to the method of Xu and colleagues (2012), with minor modifications. For determination of total hexoses, 80 μl diluted sample was added to 160 μl of 0.2% (w/v) anthrone in concentrated H_2_SO_4_, mixed well and incubated in boiling water for 5 min. After cooling down, 200 μl of the mixture was removed to the 96‐well microtiter plate and the absorbance was determined at 620 nm using a microplate spectrophotometer (Epoch; Bio Tek Instruments). For determination of total pentoses, 75 μl diluted sample and 10 μl of 6% orcinol (0.6 g orcinol dissolved in 10 ml ethanol) were mixed with 150 μl of 0.1% (w/v) ferric chloride in concentrated HCl, mixed well and incubated in the boiling water for 20 min. After cooling down, 200 μl of the mixture was transferred to the 96‐well microtiter plate and the absorbance was determined at 660 nm using a microplate spectrophotometer (Epoch; Bio Tek Instruments). A range of D‐glucose (0–100 μg ml^−1^) and D‐xylose (0–40 μg ml^−1^) concentrations were used to plot the standard curves for hexoses and pentoses respectively.

### Enzymatic hydrolysis

Enzymatic hydrolysis was performed according to NREL Laboratory Analytical Procedure LAP 009. The solid residues of pretreatment and untreated *Miscanthus* biomass were loaded at a solid concentration of 2.0% (w/v) in a 50 mM citrate buffer (pH 4.8) containing 0.005% (w/v) sodium azide. Cellulase (Celluclast 1.5L; Novozymes, Franklinton, NC, USA) from *Trichoderma reesei* and beta‐glucosidase (Novozyme 188; Novozymes, Bagsvaerd, Denmark) from *Aspergillus niger* were used for hydrolysis experiments. The enzyme loadings of Celluclast 1.5L and Novozyme 188 were 20 FPU g^−1^ glucan and 30 CBU g^−1^ glucan respectively. The hydrolysis experiment was conducted at 50°C and 150 rpm in a shaking incubator for 72 h. After enzymatic hydrolysis, the mixture was centrifuged at 12 000 g for 3 min and the supernatants were collected for measurement of hexose and pentose content. The sugars released were calculated using the following equation:

$$\text{Hexoses related}\;\left(\%\;\text{of celluose}\right)=\frac{\text{g of hexoses}}{\text{g of glucan added to hydrolysis}\times 1.11}\times 100\%$$

$$\text{Pentoses released}\;\left(\%\;\text{of hemicellulose}\right)=\frac{\text{g of pentoses}}{\text{g of xylan added to hydrolysis}\times 1.14}\times 100\%$$

$$\text{Total sugars released}\;\left(\%\;\text{of dry biomass}\right)=\frac{\text{g of hexoses}+\text{g of pentoses}}{\text{g of biomass added to hydrolysis}}\times 100\%$$

The value of 1.11 is the conversion factor of glucan to equivalent glucose. The value of 1.14 is the conversion factor of xylan to equivalent xylose. The total sugars were calculated as the total mass of glucose and xylose released treated and untreated dry biomass.

### Statistical analysis

Correlation coefficients were calculated by performing spearman rank correlation analysis. All the experiments were repeated at least three times, and the results were shown as mean ± SDs. Differences between treatments were evaluated, and statistical significance was accepted at *P* < 0.05.

## Conflict of interest

The authors declare no competing financial interest.
